# *Parasitylenchus bifurcatus* n. sp. (Tylenchida: Allantonematidae) parasitizing *Harmonia axyridis* (Coleoptera: Coccinellidae)

**DOI:** 10.1186/1756-3305-5-218

**Published:** 2012-10-01

**Authors:** George O Poinar, Tove Steenberg

**Affiliations:** 1 Department of Zoology, Oregon State University, Corvallis, OR, 97331, USA; 2 Department of Agroecology, Aarhus University, Forsogsvej 1, DK- 4200, Slagelse, Denmark

**Keywords:** Coccinellidae, Parasitic nematodes, Allantonematidae, Ladybird beetles, Multicolored lady beetle

## Abstract

**Background:**

The harlequin ladybird, *Harmonia axyridis* Pallas (Coleoptera: Coccinellidae) is native to central and eastern Asia and was purposely introduced into Europe to control aphids. While it proved to be a good biological control agent, its rapid spread and buildup of large populations made it a nuisance, since it overwinters in homes, emits unpleasant odors, stains fabrics, occasionally bites humans and feeds on apples, pears and grapes. Aside from the above, the ravenous appetite of *H. axyridis* results in their consumption of harmless native insects, including even other ladybird beetles. A study of the natural enemies of *H. axyridis* in Denmark revealed the presence of nematodes. The present study describes this nematode parasite and discusses aspects of its development and ecology.

**Methods:**

Adult harlequin ladybird beetles were collected from March to November from four localities in Copenhagen on different plant species. In addition, groups of last-instar larvae and pupae (n = 50) were examined for the presence of nematodes. Living and recently dead nematodes were removed from adult *H. axyridis* in 0.5% saline solution, the nematodes were then heat killed (at 75C), fixed in 5% formalin and transferred to glycerin on slides for further examination and measurements.

**Results:**

A new species of Allantonematidae (Tylenchida), *Parasitylenchus bifurcatus* n. sp., is described from adults of the harlequin ladybird, *Harmonia axyridis* in Denmark. The new species is characterized by a straight stylet lacking basal thickenings, a bursa and a forked tail tip in the vermiform (infective) females and juvenile males. The new species is compared with *P. coccinellinae* previously described from ladybird beetles in France. Parasitism resulted in depletion of the fat body and partial or complete atrophy of the reproductive organs of the beetles. Infections occurred throughout the year with rates of parasitism reaching up to 35%. The rate increased to 60% when field-collected ladybirds were incubated for 30 days in the laboratory.

**Conclusions:**

The production of subsequent generations within the host with only the fertilized females (not the males) leaving the hosts and the absence of parasitism of the larvae and pupae is an impressive developmental modification of *P. bifurcatus*. It is proposed that the vermiform (infective) females pass from one adult host to another when the beetles are hibernating or in assemblage groups. Rates of parasitism show that *P. bifurcatus* could be a significant biological control agent of *H. axyridis*.

## Background

The harlequin ladybird, *Harmonia axyridis* Pallas (Coleoptera: Coccinellidae) is native to central and eastern Asia. This ladybird beetle, which occurs in numerous color forms, was purposely introduced into Europe (as well as North America) to control aphids, however, while it proved to be a good biological control agent, its rapid spread and buildup of large populations made it a nuisance, since it overwinters in homes, emits unpleasant odors, stains fabrics and occasionally bites humans. Not only is it considered a household nuisance, but also a fruit pest since it feeds on apples, pears and grapes, contaminating the latter fruits to the point where wine produced from grape clusters containing adult beetles has an unpleasant flavor. Aside from the above, the ravenous appetite of *H. axyridis* results in their consumption of harmless native insects, including even other ladybird beetles [1,2]. Since the arrival of *H. axyridis* in Denmark in the mid 2000s, studies on the complex of natural enemies attacking the species have revealed the presence of a number of different parasites and pathogens [3-5], including nematodes, which were determined to be a species of *Parasitylenchus*[6]. The present study describes these nematodes as a new species, *Parasitylenchus bifurcatus*.

## Methods

**Figure 1:**
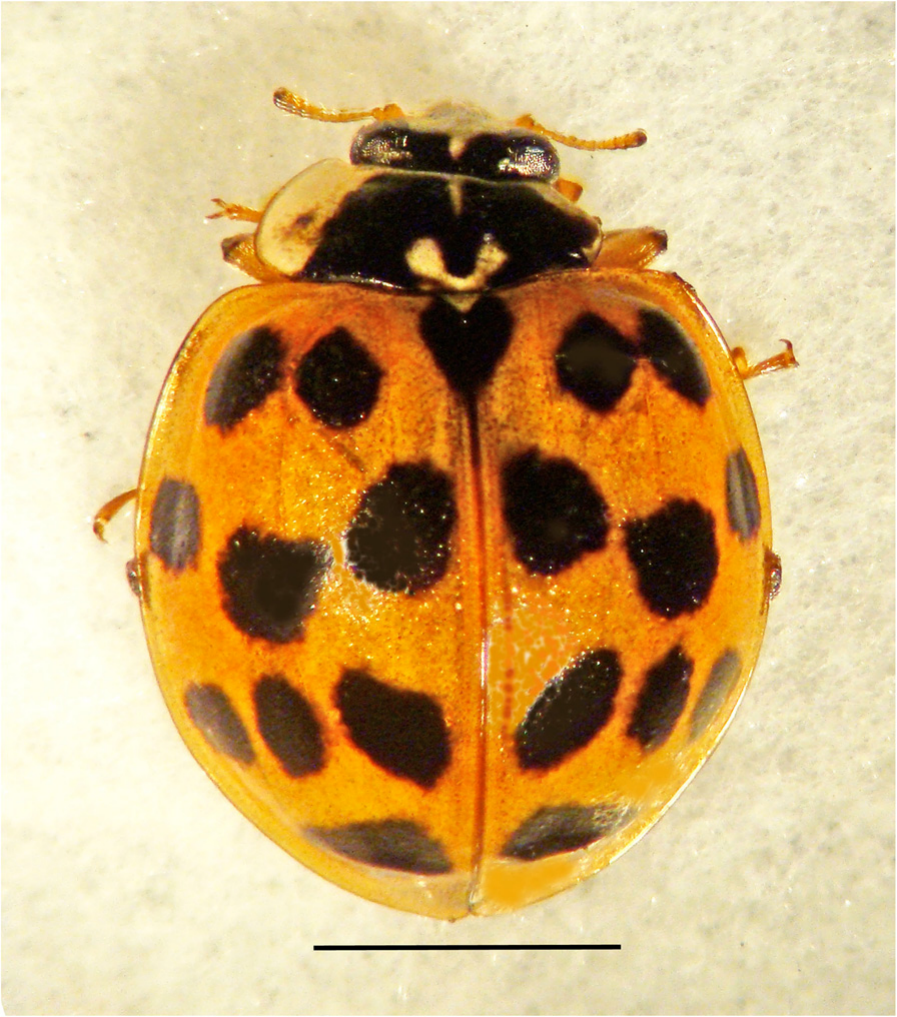
**Infected harlequin ladybird beetle, *Harmonia axyridis *(Coleoptera: Coccinellidae) from Denmark. Bar = 3 mm.**

Adult harlequin ladybird beetles (Figure 1) were collected from March to November from four localities in Copenhagen (Vanløse, Frederiksberg, Amager, Copenhagen C) on *Acer pseudoplantanus* L., *Tilia europaea* L. and *Phragmites australis* (Cav.) Trin. ex Steudel. In addition, groups of last-instar larvae and pupae (n = 50) were examined for the presence of nematodes. A group of adult *H. axyridis* (n = 118) was collected on 1 November 2010, and 20 specimens were dissected immediately after sampling. The remaining group was maintained for 30 days at room temperature in a ventilated plastic box and supplied with aphids *ad lib* and a water source. Dead specimens were dissected as well as a sample of surviving beetles (n = 20). Living and recently dead nematodes were removed from adult *H. axyridis* in 0.5% saline solution, heat killed (at 75C), fixed in 5% formalin and transferred to glycerin on slides for further examination and measurements. All measurements were made on slide mounted fixed material.

## Results and discussion

Nematode stages within the hosts included first generation parasitic females, subsequent generation parasitic females, vermiform (infective) females, and males. The vermiform females and males of all the generations had similar quantitative and qualitative characters. The value after each character is the mean and the values within the brackets represent the range.

No infections were found in last-instar larvae or pupae. At the time of collection in November 2010, the prevalence of nematodes was 35%. After 30 days of incubation, the nematode prevalence in the surviving ladybirds had increased to 60% and 11 specimens had died (11.2% of the sample incubated). Nematodes were found in 73% of these dead ladybirds.

### Description of nematode

Tylenchida Thorne, 1949

Sphaerularioidea Lubbock, 1861

Allantonematidae Pereira, 1931

*Parasitytlenchus* Micoletzky, 1922

*Parasitylenchus bifurcatus* Poinar and Steenberg, n.sp.

**Figure 2:**
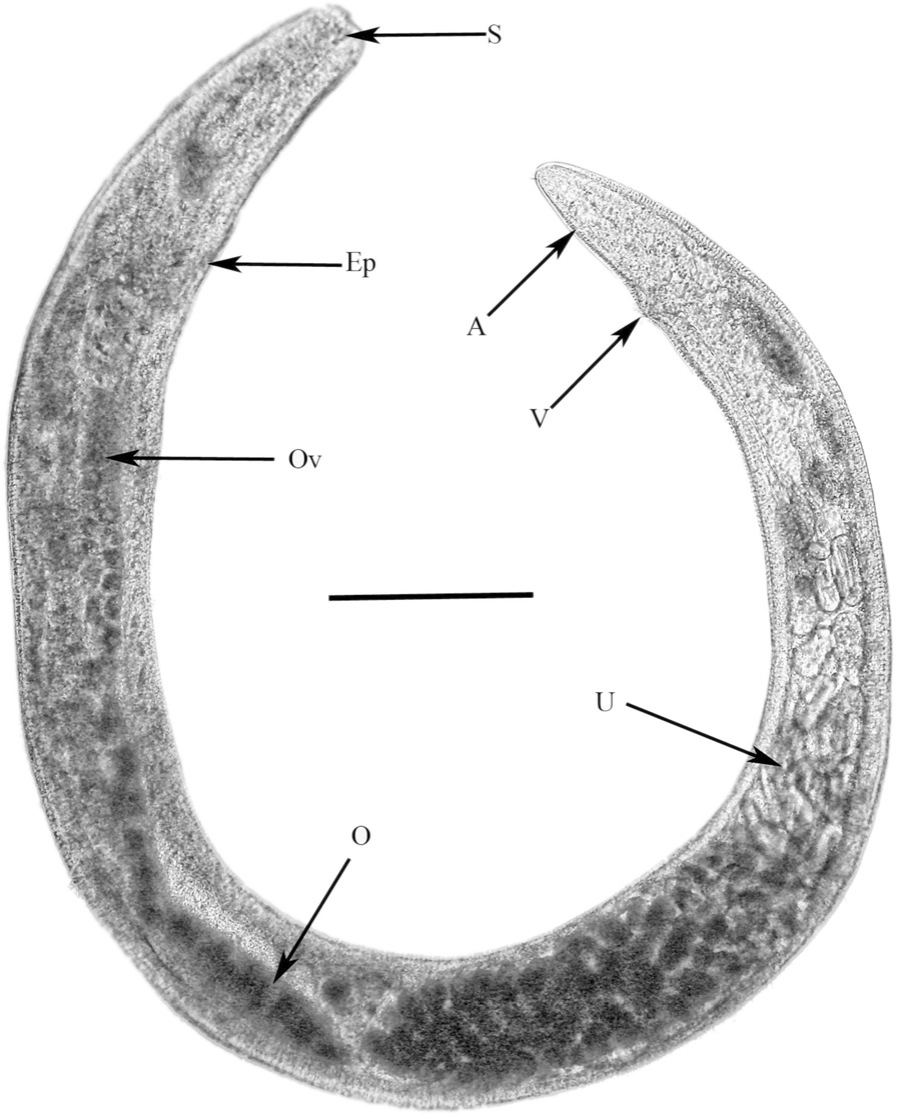
**First generation gravid female of *P. bifurcatus. ***S = stylet; Ep = excretory pore; Ov = oviduct; O = ovary; U = uterus; V = vulva; A = anus. Bar = 210 μm.

**First generation parasitic females** (n = 10)(Figure 2)

Length: 3.4 (2.5- 4.2) mm; greatest width: 158 (143-170) μm; head to excretory pore: 190 (144-300) μm; vulva to tail tip: 195 (132-224) μm; length tail: 68 (23-85) μm; % vulva: 78-95; length eggs: 64 (58-66) μm; width eggs: 28 (25-32) μm; ovoviviparous.

**Figure 3:**
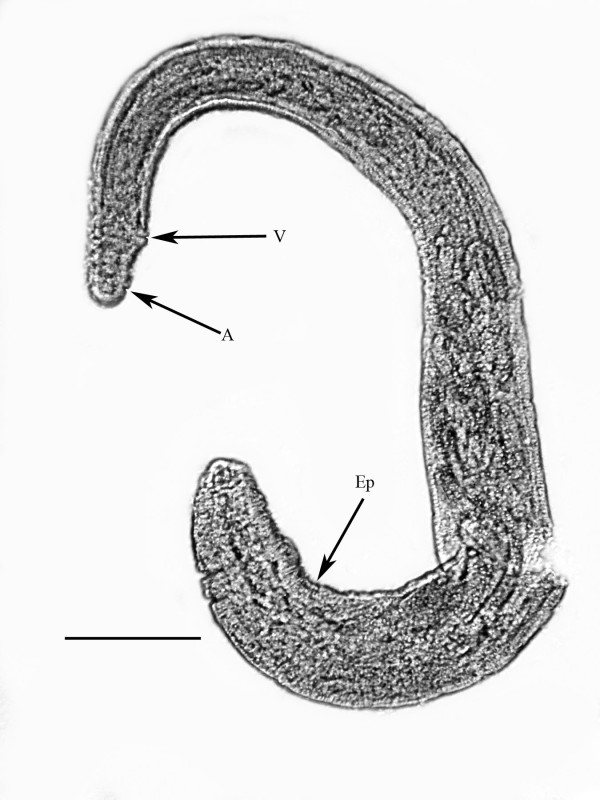
**Subseqent generation gravid female of *P. bifurcatus. ***Ep = excretory pore; V = vulva; A = anus. Bar = 124 μm.

**Subsequent generation parasitic females** (n = 10)(Figure 3)

Length:1.3 (0.92- 1.6) mm; greatest width: 195 (158-271) μm; head to excretory pore: 143 (128-158) μm; vulva to tail tip: 84 (32-195) μm; length tail: 48 (15-164) μm; % vulva: 78-97; length eggs: 47 (43-54) μm; width eggs: 25 (19-31) μm; ovoviviparous.

**Figure 4:**
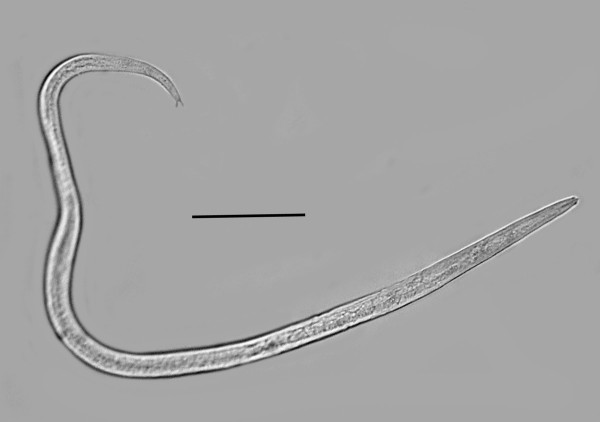
**Vermiforn (infective) female of *P. bifurcatus.*** Bar = 74 μm.

**Figure 5:**
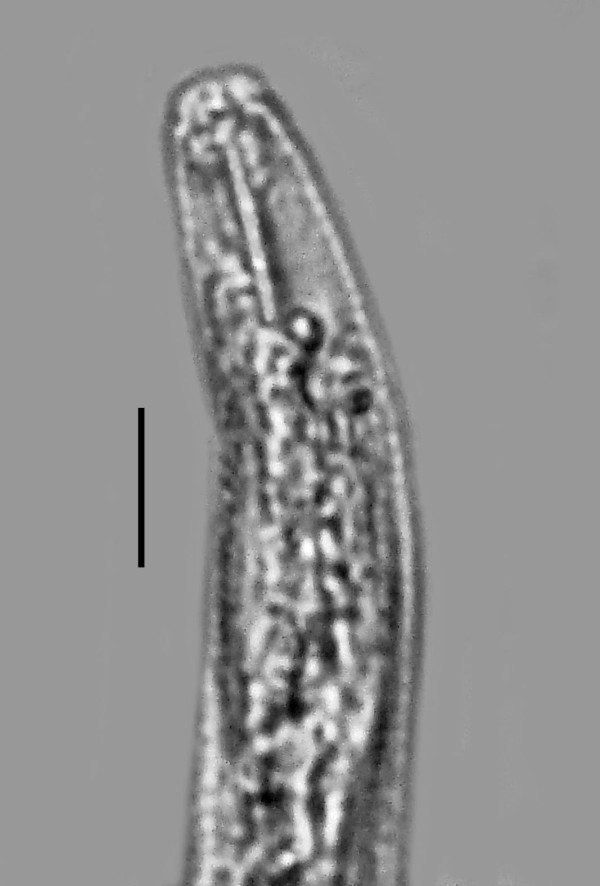
**Head of the vermiform (infective) female of *P. bifurcatus *showing stylet.** Bar = 10 μm.

**Figure 6:**
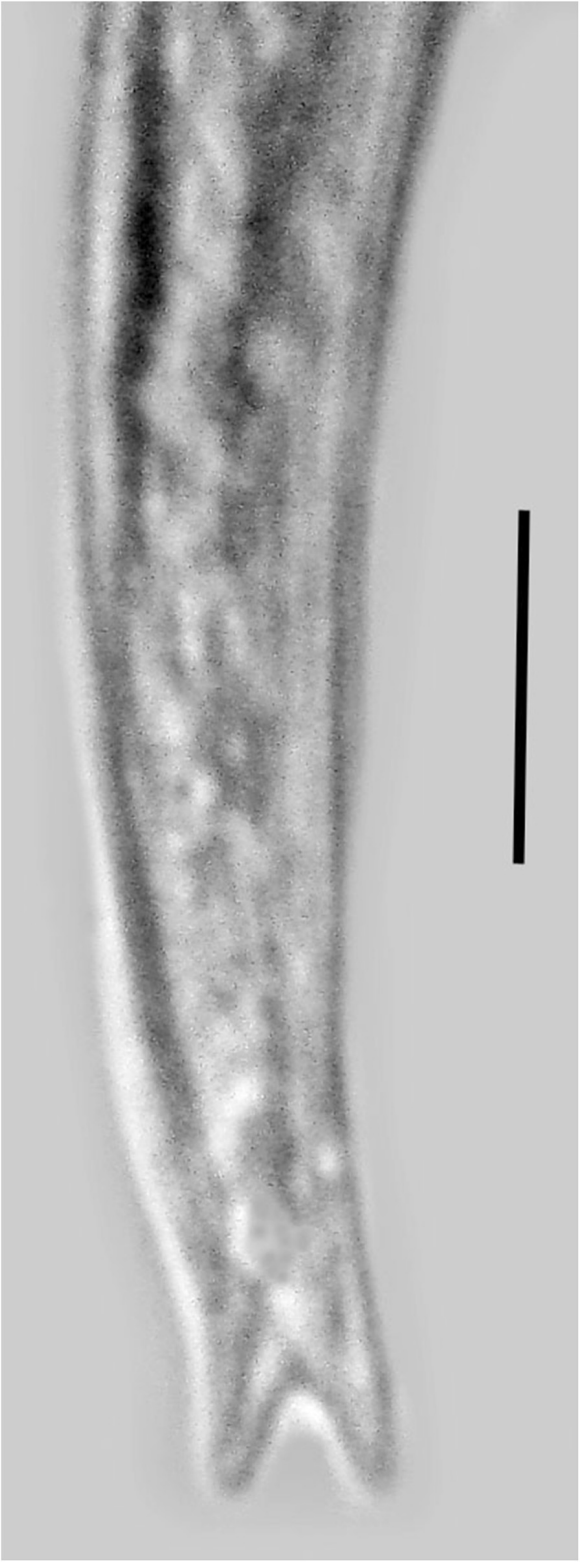
**Tail tip of vermiform (infective) female of *P. bifurcatus *showing dorsal and ventral lobes.** Bar = 9 μm

**Vermiform (infective) females** (n = 10)(Figures 4, 5, 6).

Length: 655 (592-704) μm; greatest width:14 (12-15) μm; length stylet: 10 (7-14) μm; distance from head to excretory pore: 56 (46-76) μm; distance from head to nerve ring: 52(42-68) μm; length tail: 37 (22-48) μm; vulva to tail tip: 66 (57-79) μm; vulva %: 86-95.

**Figure 7:**
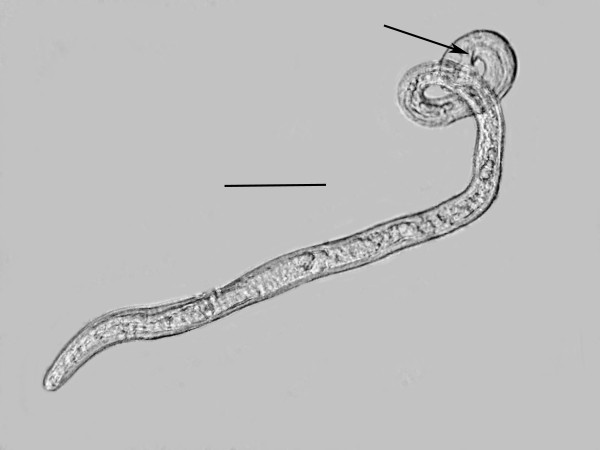
**Male of *P. bifurcatus.*** Note coiled tail region. Arrow shows spicule. Bar = 50 μm.

**Figure 8:**
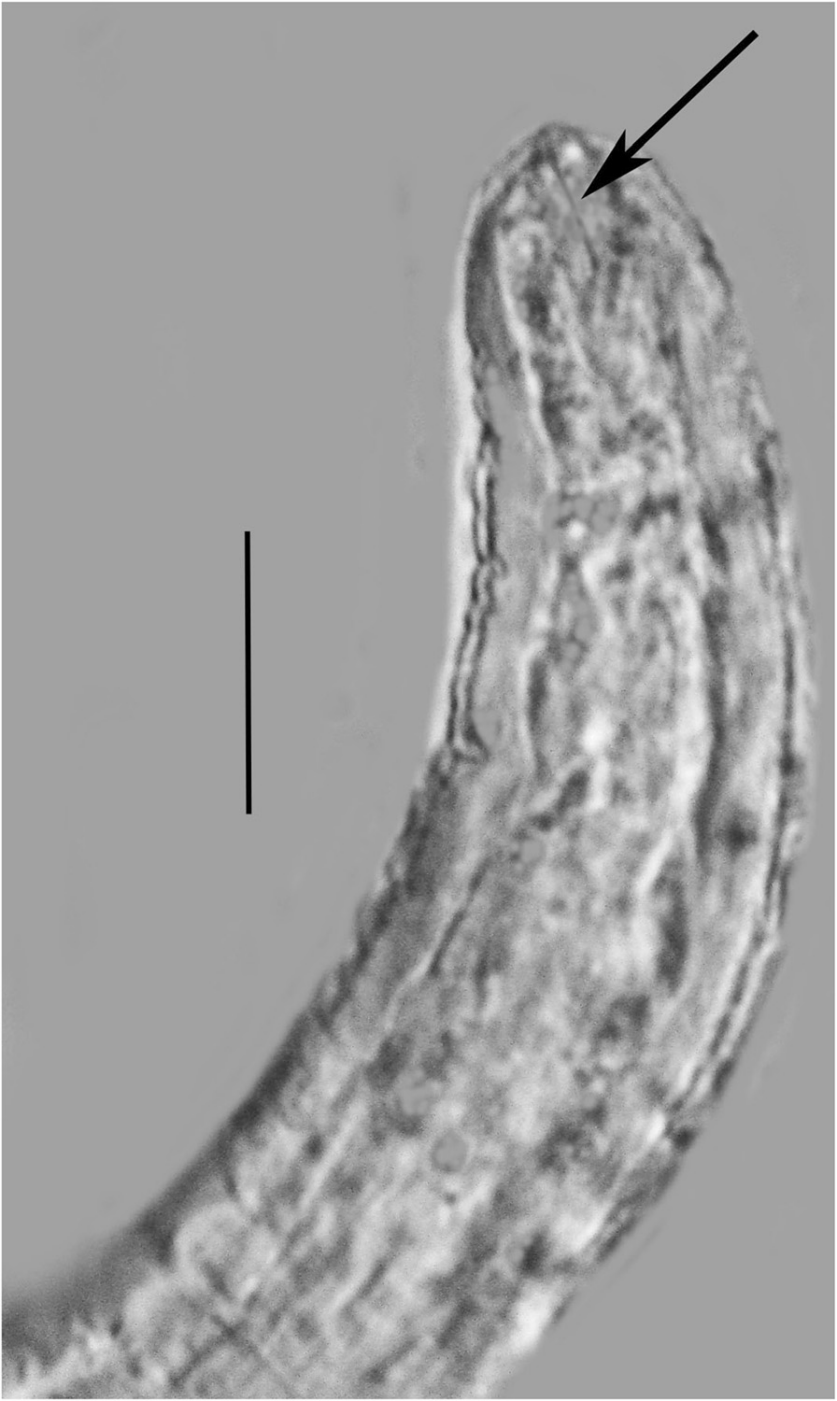
**Anterior portion of male *P. bifurcatus.*** Arrow shows stylet. Bar = 16 μm.

**Figure 9:**
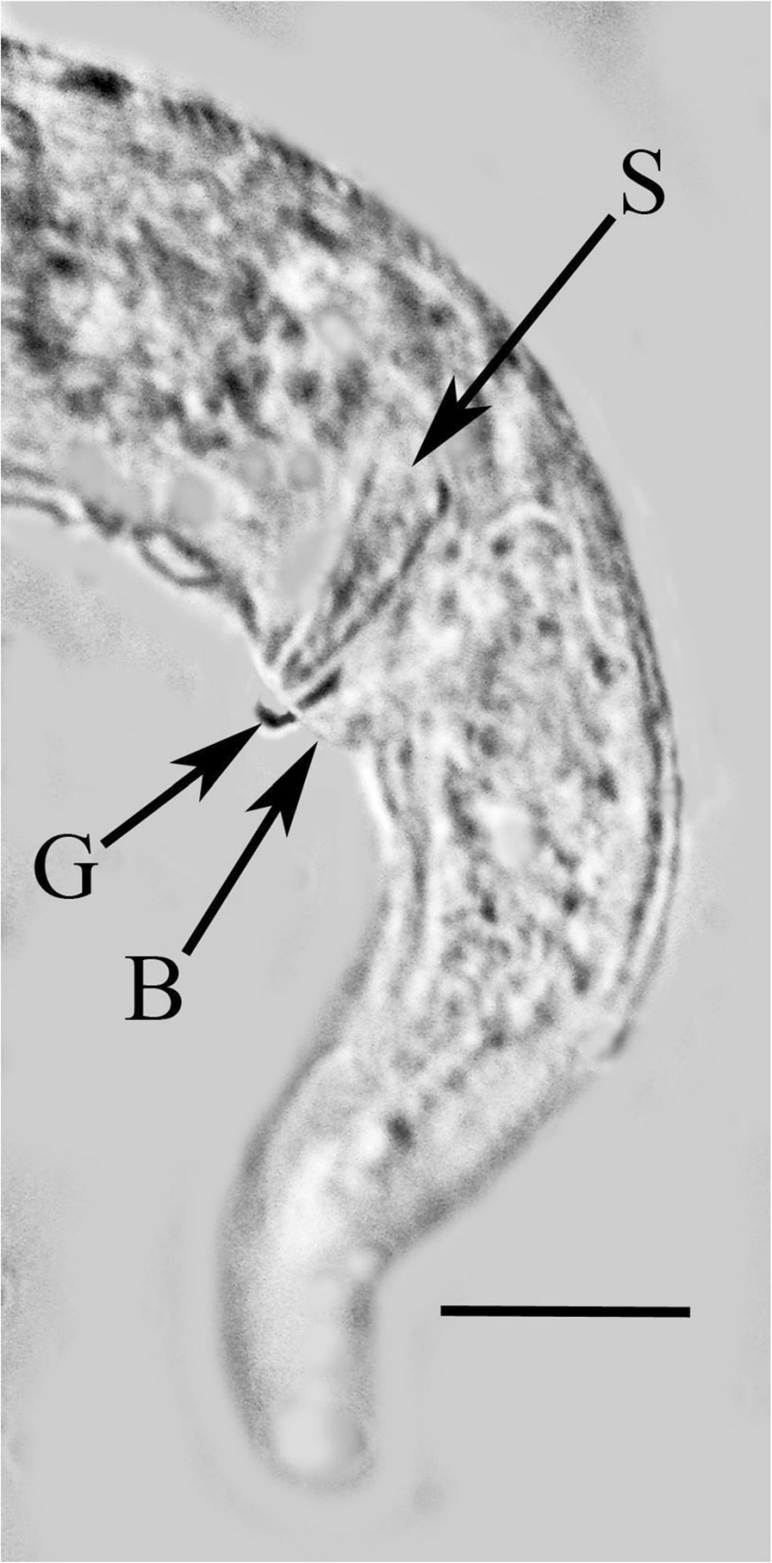
**Tail of male *P. bifurcatus.*** S = spicule; G = gubernaculum; B = bursa. Bar = 9 μm.

**Figure 10:**
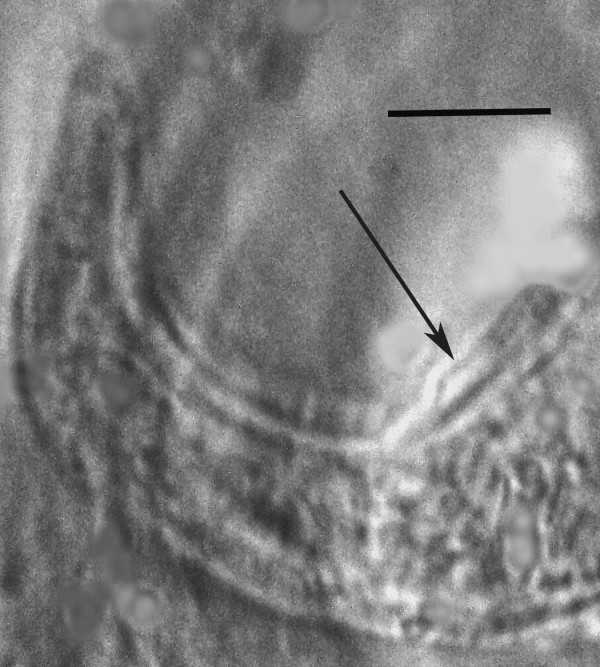
**Tail of male *P. bifurcatus *showing bursa (arrow).** Bar = 9 μm.

**Males** (n = 10)(Figures 7, 8, 9, 10).

Length: 431(357-496) μm; greatest width: 16 (14-19) μm; stylet faint, length: 8 (7-10) μm; head to excretory pore: 62 (54-73) μm; pharyngeal glands atrophied; tail tip angular-truncate, showing bifurcation in juveniles; length tail: 33 (22-40) μm; width at tail: 12 (10-14) μm; spicules straight, wedge-shaped or triangular, with wide base, length of spicules: 12 (9-14) μm; width at spicule base: 4 (3-5) μm; length of gubernaculum: 4 (3-5) μm; length of bursa: 8 (6-11) μm. The gubernaculum can assume several shapes. Usually it is straight, however it may be bent upward (Figure 9), or rarely appear double. Unfortunately the small size of this structure hinders further analysis. The bursa is short and narrow (Figures 9, 10).

The vermiform females and males have a lateral line with two incisures, however this feature is difficult to see in the parasitic females. The cuticle is very thick, especially in the vermiform females, and ridged both transversely and longitudinally. The vermiform females have an obtuse head with the lip region somewhat constricted. The straight stylet lacks basal knobs or thickenings in both sexes and the dorsal and subventral gland openings are located within three stylet lengths from the base of the stylet. The excretory pore is fairly posterior and opens opposite to or somewhat posterior to the nerve ring. The hemizonid is located just posterior to the nerve ring. The pharyngeal glands may extend to the tip of the ovary. The uterus and ovary are fairly short until the female mates, at which time the uterus expands with circular sperm cells. The great majority of vermiform females inside the host have mated. The tail tip of all vermiform females and immature males is clearly bifurcated (Figure 6).

The subsequent generations of parasitic (swollen, gravid) females tend to be shorter than the first generation forms. Young parasitic females of all generations have a relative long tail and long vulva-tail measurement. As they mature, the tail shortens, the vulva becomes more posterior in position, the stylet becomes faint, the pharyngeal glands become atrophied and the excretory pore, nerve ring, anus and vulva are more difficult to locate. The ovary is usually reflexed 2-3 times in the parasitic females.

A molt was noted inside the eggs and the body cavities of infected hosts were filled with shed cuticles of developing males and vermiform females. The vermiform females are longer and more slender than the males, which can easily be detected by their tightly coiled tail (Figure 7).

#### Diagnosis

The long, slender females lacking basal stylet thickenings but with a bifurcated tail, an excretory pore opening at the level of or somewhat posterior to the nerve ring and a gubernaculum and narrow bursa in the males separate this species from other members of the genus *Parasitylenchus*[7]. The new species is similar to *Parasitylenchus coccinellinae* Iperti & van Waerebeke [8] described from France. However the presence of a bursa and the bifurcated vermiform female tail separate the two species. Regarding the bifurcated tail of the vermiform females, it may have not been noticed by Iperti & van Waerebeke [8], who surprisingly did not give a complete description of the vermiform (infective) females. They describe the young female (presumably infective) as having a short stylet (4-5 μm) with basal thickenings, but do not characterize the tail. Basal thickenings on the stylet are lacking in *P. bifurcatus* and the stylet is 7-14 μm in length. Also Iperti & van Waerebeke [8] gave a value of 78-92 μm for the distance from the head to the excretory pore of the vermiform female, while that value ranges from 46-76 μm in *P. bifurcatus*. Concerning the males, Iperti & van Waerebeke [8] show the spicule in lateral view as curved with the base set off from the middle and apical portions, not straight and wedge-shaped as in *P. bifurcatus.* Also they give a value of 1.5 μm for the length of the male stylet, while the stylet of the male of *P. bifurcatus* ranges from 7-10 μm (Figure 6).

In their report of *P. coccinellae* in two native Indian hosts, *Menochilus sexmaculatus* (Fab.) and *Illeis indica* Timb., Reddy & Rao [9] noted a bifurcated tail in the mated vermiform (infective) females, similar to that in *P. bifurcatus*. They also characterize their populations as having a straight stylet lacking basal thickenings, which is also similar to the condition in *P. bifurcatus*. However males from the Indian population lack a bursa, the spicules are not wedge-shaped, but resemble those of *P. coccinellinae,* and the length of the vermiform females range from 400-482 μm, which is considerably shorter than those of *P. bifurcatus* (611-704 μm).

#### Etymology

The specific epithet is taken from the Latin”furcatus” = forked in reference to the cleft tail tip of the vermiform females and immature males.

#### Type host and locality

Found in the hemocoel of *Harmonia axyridis* (Pallas, 1773)(Coleoptera: Coccinellidae) in Denmark.

#### Type material

Holotype: vermiform female (T-657 t) and Paratype: parasitic female (T-6181p) deposited in the USDA Nematology Laboratory, Beltsville, Maryland. Paratypes in the authors’ collections.

## Conclusions

The genus *Parasitylenchus* is diagnosed as having three types of adults inside the host, a first generation parasitic female, a second generation parasitic female and a male [7]. Most species in the genus have minute basal knobs on the stylet. Eggs produced by the first generation females develop into juveniles that mature to adults, mate and bear eggs and juveniles inside the host. Normally, juveniles from the second generation females exit the host and the final molt and mating occurs in the environment where the infective females find new hosts. In the intertidial allantonematid, *Halophilanema prolata* Poinar infecting the hemipteran, *Saldula laticollis* (Reuter), it is the third stage juveniles that leave the host, mature and mate in the environment, with the males dying and the mated females searching for a new host [10].

In *P. bifurcatus*, as well as in the Indian strain of *P. coccinellinae *[9], there can be subsequent generations within the same host and only the fertilized females and not the males leave the hosts. Whether conditions were different, or Iperti and van Waerebeke [8] just noted a rare event when they said that after mating, both sexes of *P. coccinellinae* left the host with the males dying and the females searching for a new host, is unknown. The only reason males would leave the host would be to inseminate the exited females, and since the females have already been impregnated within the host, there is no need for the males to enter the environment.

The production of multiple generations within the host is an impressive developmental modification for *P. bifurcatus*. Another unique character of both coccinellid-parasitizing species is that host larvae and pupae are not included in the life cycle. With a continuous series of generations, large nematode populations can be formed inside the beetles, especially the females where ovarian tissue is utilized by the parasites. Iperti and van Waerebeke [8] reported a single adult coccinellid with 140 mature females and more than 10,000 juveniles and vermiform adults. Reddy and Rao [9] noted that up to four nematode generations could occur in a single beetle. Obviously, all of the vermiform females formed within the host cannot initiate development in the same beetle and the availability of nutrients probably determines how many generations are produced. When the limit has been reached, the vermiform (infective) females prepare to leave the host and seek another one.

How the nematodes find and enter uninfected adult beetles is unknown. However, Reddy and Rao [9] noted that some infective females exited through the host’s ovipositor. A natural infection location would be hibernation or aestivation sites of the adult beetles. *H. axyridis* responds to β-caryophyllene, which acts as an aggregation pheromone [11], and t his is likely to increase the likelihood of parasite transmission.

The infective stages could emerge from one beetle and directly enter another without much searching and without much exposure to the environment. The thick cuticle on the vermiform (infective) females would definitely be a survival factor once they exited the host.

Penetration into the new host is probably aided by secretions from the pharyngeal glands and action of the well-developed stylet, as in other species of Allantonematidae. Infection by vermiform females in the environment is supported by the discovery of infective females in debris from assemblage locations of adult beetles [9]. The ladybird species reported to be parasitized by *Parasitylenchus* are mostly multivoltine and thus have overlapping generations, which also is likely to aid parasite transmission. The demonstrated increase in infection rates from 35% to 60% within 30 days in a group of *H. axyridis* shows the transmission potential of *P. bifurcatus* no matter what the exact mode of transmission may be. Another possibility is that the females are transferred from host to host during mating, thus eliminating the need for infective stage females from exiting the host.

Rates of parasitism in *H. axyridis* collected in the autumn and early winter were reported to vary from 2 to 33.3% [6]. Subsequent dissection of *H. axyridis* sampled throughout the year from March to November showed that in 2011 the nematodes were present all through the year (Steenberg, unpublished data). The effect of the nematodes on host populations is difficult to assess, however both Iparti & van Waerebeke [8] and Reddy & Rao [9] noted that the nematodes depleted the fat body and reproductive organs of the host. Similar effects were noted with *P. bifurcatus*. Both male and female beetles were parasitized [6], and although more detailed studies are lacking, similar parasites often have severe consequences on host fitness, including female sterility, reduced survival and lower male fertility and mating success [12]. When maintained under stress with reduced food and water, infected *H. axyridis* adults were the first to succumb. We discovered that 73% of *H. axyridis* adults dying over a 30 day incubation period were parasitized by *P. bifurcatus*, which shows that the nematodes may indeed be a significant mortality factor of *H. axyridis*. However, its main effect on ladybird populations may be sublethal, since infection rates of 35% caused slightly over 8% direct mortality over the 30 day incubation period.

The original source of infection in *H. axyridis* is unknown. While it could have originated with parasitized individuals introduced from Asia into Europe, or via naturalized populations accidently introduced from North America into Europe, it is possible that the infections were acquired from endemic European ladybird beetles, such as *Oenopia conglobata* (L.) and other species in France [8] and *Adalia bipunctata* (L.) in the UK [13]. It is likely that various species and strains of *Parasitylenchus* parasitize coccinellids wherever these beetles occur. Genomic studies will help to determine how closely related these populations are.

In accordance with section 8.6 of the ICZN’s International Code of Zoological Nomenclature, copies of this article are deposited at the following five publicly accessible libraries: Natural History Museum, London, UK; American Museum of Natural History, New York, USA; Museum National d’Histoire Naturelle, Paris, France; Russian Academy of Sciences, Moscow, Russia; Academia Sinica, Taipei, Taiwan.

## Competing interests

The authors declare that they have no competing interests.

## Authors’ contributions

TS discovered the nematode and provided biological data. GP wrote the description and supplied the figures. Both authors read and approved the final version of the manuscript.
